# Impact of colonoscopy on health-related quality of life: findings from the RECEDE study

**DOI:** 10.1186/s12955-024-02262-x

**Published:** 2024-06-26

**Authors:** L. Andronis, N. Waugh, M. Zanganeh, A. Krishnamoorthy, N. Parsons, M. Hull, P. Wheatstone, R. P. Arasaradnam, On behalf of the RECEDE study group

**Affiliations:** 1 Warwick Evidence, University of Warwick, Coventry, UK; 2 Clinical Trials Unit, Warwick Medical School, University of Warwick, Coventry, UK; 3 Institute of Applied Health Sciences, University of Aberdeen, Aberdeen, UK; 4 Dept of Molecular Medicine, University of Leeds, Leeds, UK; 5 Patient and Public representative, University of Leeds, Leeds, UK; 6 Institute of Precision Diagnostics & Translational Medicine, UHCW, Coventry, UK; 7 Leicester Cancer Centre, University of Leicester, Leicester, UK; 8 Health Sciences, Warwick Medical School, University of Warwick, Coventry, UK

## Abstract

**Background:**

Colonoscopy is a valuable diagnostic tool but the procedure and the preparation for it cause anxiety and discomfort that impacts on patients’ health-related quality of life (HRQoL). The ‘disutility’ of undergoing an invasive colonoscopy needs to be considered and accounted for in comprehensive cost-utility analyses that compare different diagnostic strategies, yet there is little empirical evidence that can be used in such studies. To fill this gap, we collected and analysed data on the effect of a colonoscopy examination on patients’ HRQoL that can be used in economic evaluations.

**Methods:**

Patients scheduled to undergo a colonoscopy at a large NHS hospital were asked to complete the EuroQol EQ-5D-5 L instrument: (i) before the procedure, at the time of consent (T1), (ii) while undergoing bowel preparation (T2) and (iii) within 24 h after the procedure (T3). Complete responses were translated into preference-based HRQoL (utility) values using a UK-specific value set and were analysed using descriptive and inferential statistical analyses.

**Results:**

Two-hundred and seventy-one patients with gastrointestinal symptoms referred for a colonoscopy provided complete EQ-5D-5 L questionnaires at all three assessment points. At T1, the mean EQ-5D-5 L value was 0.76 (95%CI: 0.734–0.786). This value dropped to 0.727 at T2 (95%CI: 0.7–0.754, before increasing again to 0.794 (95%CI: 0.768–0.819) at T3. Both changes were statistically significant (p-value < 0.001).

**Conclusions:**

Preference-based HRQoL (utility) values reported by patients undergoing a colonoscopy dropped during bowel preparation and rose again shortly after the colonoscopy. This pattern was largely consistent across patients with different characteristics, symptoms and diagnoses.

**Supplementary Information:**

The online version contains supplementary material available at 10.1186/s12955-024-02262-x.

## Introduction

Every year, in the UK alone, more than 350,000 patients experiencing lower gastrointestinal symptoms such as change in bowel habit, rectal bleeding, or iron deficiency anaemia are referred to a hospital for an invasive colonic examination, most often a colonoscopy [1]. While colonoscopy is a valuable diagnostic tool, the procedure and the bowel preparation for it causes anxiety and discomfort [2]. Patients have to alter their diet 48 h before their colonoscopy, stop certain medications and fast for 12 h prior the procedure. This has an impact on patients’ lifestyle, especially in those with diabetes and the elderly [3], while patients with renal disease have to take a modified form of bowel preparation to avoid kidney injury. Not surprisingly, patients find the bowel preparation that is required prior to colonoscopy very intrusive–often more so than the procedure itself [4].

Such concerns, coupled with an increasing demand and limited capacity for colonic examinations have led to a strong interest in effective ways of identifying bowel disease and dependable tools for prioritising and avoiding unnecessary examinations [5]. This has given rise to diagnostic and triage approaches which, prior to wider adoption, need to be assessed in economic evaluations [5–8]. In order to make findings useful for decision-making, outcomes of economic evaluations are often presented in terms of quality-adjusted life years (QALYs) [9]. To calculate QALYs, it is necessary to know how different components of a care pathway, including diagnostic procedures, impact on a patient’s health-related quality of life (HRQoL), with the latter being expressed in the form of a preference-based metric [10]. Yet, there has been a lack of usable estimates of the (temporary) decrement in HRQoL associated with undergoing the procedure. In the absence of such information, this decrement is inevitably and pragmatically approximated by using assumptions. For example, in a recent study, the authors explain *“We found no available estimate on the negative impact on health-related quality of life of colonoscopy or its adverse events. Therefore, we limited our analysis to explore the effect of a disutility of colonoscopy of 0.0075 in the month when referral takes place, that is the equivalent of a loss of life in full health of 5 hours.”* [6] In other cases, the ‘disutility’ associated with the procedure is not included in calculations thus, in effect, the temporary decrease in HRQoL is missed out altogether [7].

To address this gap, we collected and analysed data on the (temporary) impact of a colonoscopy examination on patients’ HRQoL using a widely used validated instrument that enables the calculation of preference-based HRQoL (utility) values and QALYs.

## Methods

### Study participants, procedure and data collection

Patient recruitment and data collection were undertaken within the context of the RECEDE study, funded by the National Institute for Health and Care Research in the UK (reference: NIHR127800). The study and its primary aims are described in detail in the study’s protocol [11]. In brief, RECEDE investigates whether the combination of faecal immunochemical testing (FIT) and volatile organic compounds (VOC) analysis improves detection of significant bowel disease (SBD) and avoid unnecessary colonoscopy examinations in patients who present with lower gastrointestinal (GI) symptoms, compared with FIT alone.

Study participants comprised consenting RECEDE patients recruited at University Hospital Coventry and Warwickshire (UCHW), a large NHS hospital in West Midlands, UK. Patients were eligible to take part in RECEDE if: (i) they presented lower gastrointestinal symptoms and were referred, either routinely or urgently, for colonic investigation by their overseeing clinician; (ii) were older than 18 years of age and, (iii) were able to provide informed consent. Colonoscopy—an examination of the large bowel (colon) using a flexible tube with a camera and light source at the end—comprised preparation and the actual procedure. The preparation phase started 48 h before the test itself with the intention of purging the colon of its contents to facilitate adequate visualisation of the lumen (bowel preparation), initially with a low-residue diet and then use of a strong osmotic laxative 24 h before the procedure. The majority of bowel preparations at UCHW used Moviprep™(macrogol 3350) but the stimulant laxative Picolax™ was used in a few cases. On the day of the procedure, individuals were offered the option of light sedation with midazolam and/or fentanyl or the use of a 1:1 nitrous oxide/air mix (Entonox). The procedure time is variable depending on several factors but is usually completed within 30–40 min. A written report is provided to the individual on the same day, but they may need to wait a few weeks longer to get the results from specimens sent to the lab for histopathological assessment.

The study presented here was pre-specified in the funding application and research protocol for RECEDE. As part of the study, patients were asked to complete a short questionnaire asking questions about their health at three predetermined points in time (assessment points): (i) T1: before colonoscopy, at time of consent to study participation (baseline), (ii) T2: within 24 h prior to colonoscopy, during bowel preparation, and (iii) at T3: within 24 h after colonoscopy. In addition, information was available for participants’ characteristics (e.g., age, weight, heigh, ethnicity, smoking status, alcohol consumption), symptoms (e.g., passing of liquid stools, blood in stools, mass in abdomen) and colonoscopy findings (e.g., presence of cancerous lesion, Crohn’s disease, ulcerative colitis etc.). Findings were grouped as diagnosis of significant bowel disease (SBD) if the colonoscopy identified either a cancerous lesion, Crohn’s disease, ulcerative colitis or polyps > 10 mm) and no SBD otherwise. Ethics approval was obtained from the North West - Liverpool Central Research Ethics Committee (25/08/2022; reference: 20/NW/0346).

### Health-related quality of life instrument

Participants’ HRQoL was measured through the EuroQol EQ-5D-5 L, a validated and recommended generic measure of HRQoL that enables the calculation of preference-based (utility) values and QALYs [12]. The EQ-5D-5 L comprises a descriptive system and a visual analogue scale (EQ VAS) [13]. The descriptive system asks respondents to indicate the level corresponding to their current health state (no problems, slight problems, moderate problems, severe problems, unable to/extreme problems; coded as 1,2,3,4 and 5, respectively) under each of five dimensions (mobility, self-care, usual activities, pain/discomfort, anxiety/depression). A respondent’s answer to the descriptive system corresponds to a unique EQ-5D-5 L profile presented as a five-digit number (e.g., 12,134). The EQ VAS asks respondents to indicate their self-rated health by placing a mark on a vertical scale numbered from 0 to 100, with endpoints labelled as ‘The worst health you can imagine’ (corresponding to 0) and ‘The best health you can imagine’ (corresponding to 100).

Each EQ-5D health profile is translated into a unique EQ-5D-5 L value (often called a utility value) using a value set, comprising a set of preference weights. EQ-5D values aims to reflect the population preference for health states and are used in the calculation of QALYs. To convert EQ-5D-5 L profiles to values, we used the crosswalk approach described in Hernandez Alava et al. [14], which is, at the time of writing, recommended by the National Institute for Health and Care Excellence in the UK [9].

### Statistical analyses

Information collected from participants with complete data were analysed using different approaches, including crosstabulation of categorical or ordinal variables as well as calculation of descriptive statistics (means and medians values, proportions, Pearson’s correlation scores) and inferential statistics (regression analysis and hypotheses tests). Standard errors and confidence intervals were generated through non-parametric (bias corrected and accelerated) bootstrapping [15]. Analyses were carried out in Stata 18 (StataCorp, College Station, TX, USA) and were guided by recommendations on methods for analysing and presenting HRQoL information [16]. P-values were considered statistically significant if they were equal to or lower than 0.05.

## Results

### Respondent characteristics

Four-hundred and sixty-seven eligible participants consented to take part in this study by providing HRQoL data. Of those, two patients withdrew from the study before any of their data was recorded, four patients withdrew during the study, five patients did not undergo an endoscopic procedure, 14 patients underwent a procedure other than colonoscopy (computed tomography colonography) and two participants did not completed their colonoscopy examination. Of the 440 participants who had a completed colonoscopy, 271 provided complete EQ-5D-5 L data at the predetermined three assessment points. These participants comprised the main sample of the study; their characteristics are shown in Table 1.

**Table 1:** Demographic characteristics

	Respondents (*n* = 271)^a^
**Age (mean)**	60.62
**BMI (mean)**	27.89
**BMI group (%)**	
Healthy weight	28.78%
Underweight	3.70%
Overweight	41.70%
Obese	29.15%
**Sex (%)**	
Male	47.60%
Female	52.40%
**Ethnicity (%)**	
White British	94.46%
White Irish	1.48%
White Other	1.85%
White and black African	0.00%
White and Asian	0.00%
Chinese	0.00%
Indian	1.11%
Pakistani	0.37%
Other Asian Background	0.37%
Caribbean	0.00%
African	0.37%
**Alcohol consumption (%)**	
Non-drinker	38.01%
Drinker	61.99%
**Cigarette smoking (%)**	
Non-smoker	67.16%
Former Smoker	22.51%
Current Smoker	10.33%
**Number of cigarettes smoked** ^**b**^	
1 to 9	22.47%
10 to 19	44.94%
20 to 29	24.72%
More than 30	7.87%
**Mass in abdomen identified (%)**	
None	98.89%
Definite	0.74%
Definite and tender	0.37%
**Instances of liquid stool passing per day (%)**	
None	36.16%
1 to 3	27.68%
4 to 5	16.97%
6 or more	19.19%
**Blood in stools (%)**	
None	51.66%
Streaks of blood with stools in less than half of cases	12.55%
Obvious blood with most of stools	10.70%
Blood alone passes	25.09%
**Anaemia (%)**	
No	79.70%
Yes	20.30%

^a^ Sample comprises patients who did not withdraw during the study, underwent a colonoscopy and provided complete EQ-5D-5 L responses at time points 1–3.

^b^ Amongst current smokers (*n* = 89).

### EQ-5D-5 L dimensions and levels

**Fig. 1:**
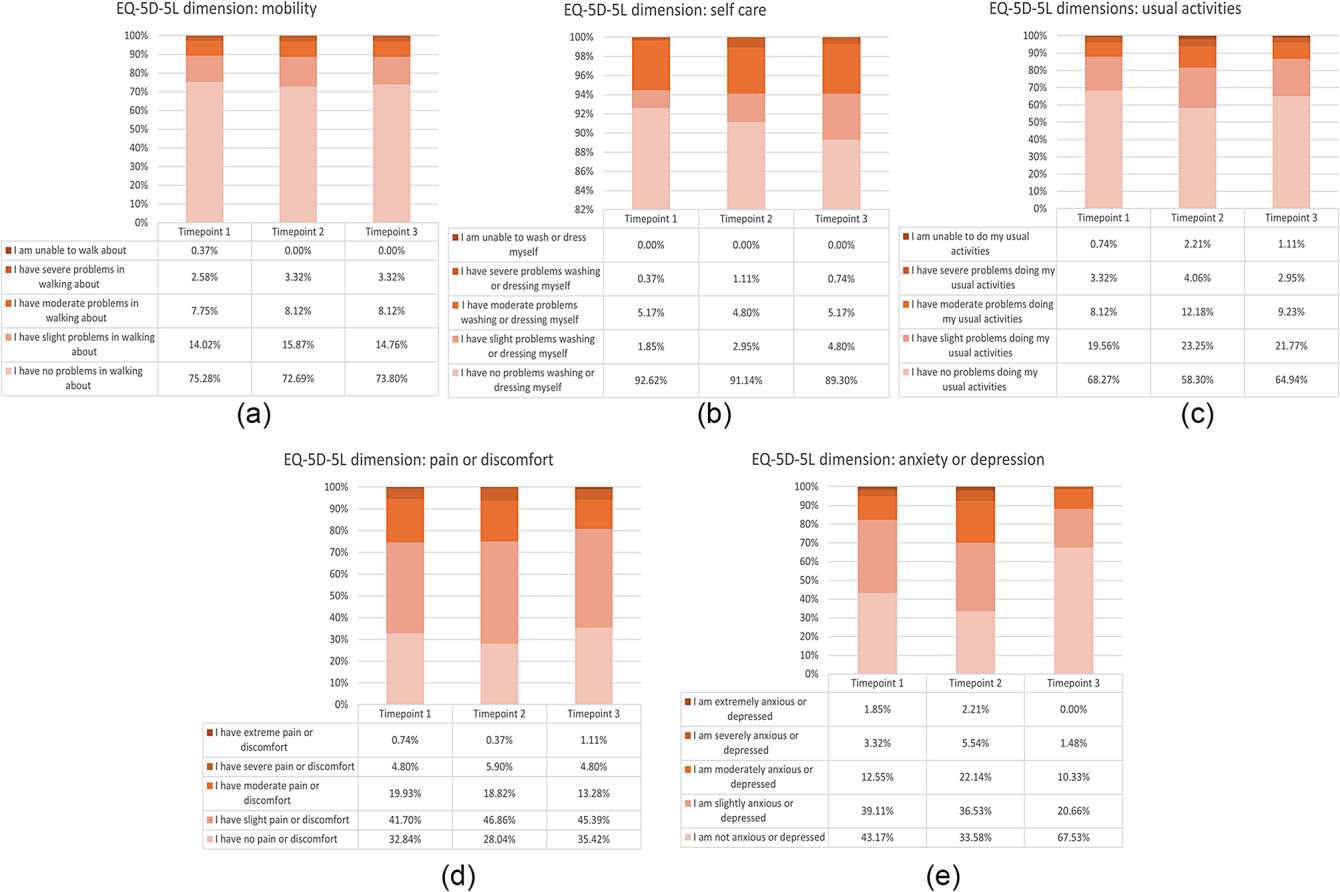
(**A**-**E**) Frequency of reported levels by EQ-5D-5 L dimension

Responses to the health status description part of the EQ-5D-5 L are summarised in Fig. 1. Each graph shows one of the five EQ-5D dimensions and, within each dimension, stacked bars show the frequency by which respondents indicated different levels.

Most respondents reported no problems with ‘mobility’, ‘self-care’ and ‘usual activities’, and this pattern held across all three time points. Notably, approximately 90% of all respondents indicated no problems with ‘self-care’ before, during and after colonoscopy. On the other hands, most respondents indicated at least some problems with ‘pain/discomfort’ and ‘anxiety/depression’. Specifically, at T1 (i.e., at time of consent), approximately 6% of the respondents reported severe or extreme ‘pain/discomfort’ and 5% reported severe or extreme ‘anxiety/depression’. At T2 (i.e., during bowel preparation), a greater number of respondents reported some problems with pain/discomfort (either slight, moderate, severe or extreme anxiety/depression reported by 72% compared to 67% at T1, *p* = 0.22) and markedly more respondents reported problems with anxiety/depression (either slight, moderate, severe or extreme ‘anxiety/depression’ reported by 66% compared to 57% at T1, *P* = 0.02). At T3 (after colonoscopy), responses to the dimension ‘anxiety/depression’ showed a notable change compared to T2, with the number of respondents reporting ‘no problems’ more than doubling (from 91 at T2 to 183 at T3, p < 0.001), and the number of people reporting severe or extreme problems being reduced by about 80% (from 15 at T2 to 4 at T3, p = 0.01).

**Fig. 2:**
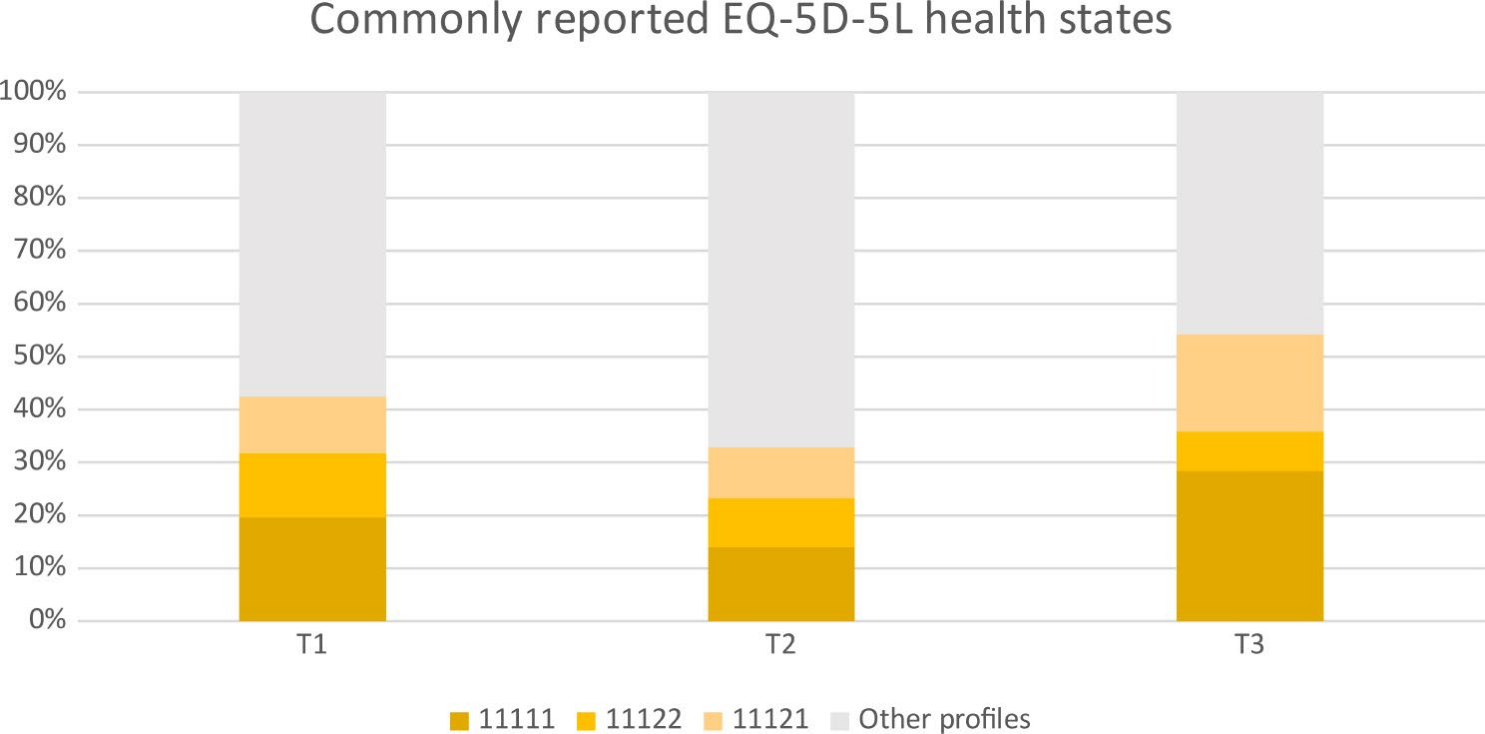
Most frequently reported EQ-5D-5 L profiles

Nearly 20% of the respondents at T1 reported ‘no problems’ in all dimensions (coded as 11,111), which was the most frequently reported state, followed by the states ‘some problems with pain/discomfort and some problems with anxiety/depression’ (11,122, reported by 12%) and ‘some problems with pain or discomfort’ (11,121, reported by 11%). Responses were similar at T2: the commonest states were the ‘no problems’ state, followed by ‘some problems with pain or discomfort’ and ‘some problems with pain or discomfort and some problems with anxiety or depression’, reported by 14%, 10% and 9% of all respondents, respectively. Time point 3 saw a substantial increase in the number of people who reported ‘no problems’ and ‘some problems with pain or discomfort’, to 28% and 18%, respectively, with comparatively fewer patients reporting ‘some problems with pain or discomfort and some problems with anxiety or depression’ (7%) (Fig. 2).

### Visual analogue scale

Summary statistics for participants’ responses to the EQ VAS can be seen in the upper half of Table 2. Across time points, the mean EQ VAS score was consistently above 70, starting at over 76, then falling to less than 74 at time point 2 and rising again to its highest mean value of nearly 78. Both the change from T1 to T2 (reduction by 2.8 scale points), and from time T2 to T3 (increase by 4.3 points) were statistically significant (*p* < 0.001). EQ VAS values at different time points were positively and statistically significantly correlated (*p* < 0.001) with each other. For example, higher EQ VAS scores at T1 were associated with higher EQ VAS scores at subsequent time points (see Supplementary Material, Table 1).

**Table 2:** Summary statistics for EQ VAS responses and EQ-5D values

	T1	T2	T3	Δ (T2-T1)^a^	Δ (T3-T2) ^a^
EQ-VAS (*n* = 271)					
Mean	76.4	73.6	77.9	-2.8	4.3
SE^b^	17.1	18.7	18.1	0.61	0.66
Lower 95% CI^b^	74.3	71.4	75.7	-4.0	3.0
Upper 95% CI^b^	78.4	75.8	80.0	-1.6	5.6
Min	20	10	10	-55.0	-40
Max	100	100	100	36	58
p-value				0.000	0.000
**EQ-5D-5 L index**(*n*** = 271**)					
Mean	0.760	0.727	0.794	-0.033	0.067
SE^b^	0.214	0.227	0.214	0.007	0.008
Lower 95% CI^b^	0.734	0.700	0.768	-0.047	0.051
Upper 95% CI^b^	0.786	0.754	0.819	-0.019	0.083
Min	-0.163	-0.187	-0.147	-0.493	-0.316
Max	0.989	0.989	0.989	0.607	0.708
p-value				0.000	0.000

^a^ Difference in mean scores between T2 and T1, and T3 and T2

^b^ Calculated using bias corrected and accelerated bootstrapping

### EQ-5D values

Summary statistics for EQ-5D values can be seen in the lower half of Table 2. The mean EQ-5D values at T1 was 0.76. At T2, this decreased to 0.73, registering a statistically significant reduction by 0.03 units (95% CI: -0.047 to -0.019, *p* < 0.001), before rising to 0.79 at T3, showing a statistically significant increase by 0.07 (95% CI: 0.051 to 0.083, *p* < 0.001). EQ-5D-5 L values were positively and significantly correlated with EQ VAS responses registered at the same points in time (see Supplementary Material, Table 1).

EQ-5D-5 L at different time points reported by participants with different characteristics and symptoms are presented in Table 3. Looking across time points, differences in values reported at T1 and T2 were prominent and highly statistically significant at *p* < 0.000 for females, White British, classified as obese, non-smokers, those who smoked or smoke more than 30 cigarettes a day, those with no mass found in their abdomen and no anaemia. Between T1 and T2, differences in EQ-5D-5 L values were statistically significant (*p* < 0.001) for respondents between 70 and 69 years old, White British, of healthy weight, non-smokers, with no mass in their abdomen, having up to 3 instances of liquid stool passing a day, with no rectal bleeding, no anaemia and diagnosed with a condition that is not immediately life threatening.

**Table 3:** EQ-5D values by respondents’ demographic characteristics

	*N*	T1		T2		T3		Diff (T2 – T1)		Diff (T2 – T3)	
EQ-5D-5 L index values		**Mean**	**SD**	**Mean**	**SD**	**Mean**	**SD**	**Mean**	**p-value** ^**a**^	**Mean**	**p-value** ^**a**^
**Age group**											
20–39	17	0.732	0.164	0.678	0.244	0.791	0.145	-0.054	0.105	0.113	0.027*
40–59	101	0.761	0.229	0.726	0.220	0.784	0.224	-0.034	0.012*	0.058	0.000***
60–79	147	0.759	0.211	0.730	0.232	0.799	0.218	-0.029	0.001***	0.069	0.000***
over 80	6	0.852	0.130	0.803	0.173	0.835	0.112	-0.049	0.088	0.032	0.556
**Sex**											
Female	142	0.708	0.251	0.662	0.257	0.736	0.255	-0.046	0.000***	0.074	0.000***
Male	129	0.817	0.144	0.798	0.162	0.857	0.130	-0.019	0.026*	0.059	0.000***
**Ethnicity**											
White British	256	0.756	0.117	0.722	0.160	0.791	0.113	-0.035	0.000***	0.069	0.000***
Other ethnicity	15	0.822	0.218	0.816	0.229	0.839	0.218	-0.006	0.803	0.023	0.528
**BMI group**											
Healthy weight	78	0.778	0.182	0.753	0.203	0.829	0.189	-0.026	0.067	0.077	0.000***
Underweight	1	0.893	n/a	0.793	n/a	0.793	n/a	-0.101	n/a	0.000	n/a
Overweight	113	0.787	0.216	0.756	0.217	0.811	0.213	-0.030	0.012*	0.054	0.000***
Obese	79	0.702	0.230	0.658	0.251	0.735	0.229	-0.043	0.000***	0.076	0.000***
**Alcohol drinking**											
Alcohol drinker	168	0.792	0.189	0.761	0.191	0.823	0.191	-0.031	0.001***	0.062	0.000***
Non-drinker	103	0.708	0.241	0.671	0.267	0.746	0.240	-0.037	0.001***	0.075	0.000***
**Smoking**											
Non-smoker	182	0.771	0.193	0.734	0.212	0.806	0.194	-0.037	0.000***	0.072	0.000***
Former Smoker	61	0.804	0.166	0.765	0.176	0.819	0.185	-0.039	0.009**	0.054	0.000***
Current Smoker	28	0.589	0.332	0.596	0.351	0.657	0.327	0.008	0.827	0.061	0.047*
**Cigarettes per day** ^**b**^											
1 to 9	20	0.664	0.371	0.683	0.324	0.741	0.328	0.018	0.669	0.059	0.041*
10 to 19	40	0.733	0.218	0.688	0.254	0.745	0.244	-0.046	0.028*	0.057	0.01*
20 to 29	22	0.748	0.179	0.727	0.204	0.779	0.192	-0.021	0.062	0.052	0.059
More than 30	7	0.926	0.059	0.890	0.106	0.942	0.080	-0.035	0.000***	0.052	0.225
**Abdominal mass**											
None	268	0.760	0.215	0.727	0.227	0.794	0.215	-0.032	0.000***	0.067	0.000***
Definite	2	0.856	0.017	0.798	0.017	0.795	0.003	-0.058	0.250	-0.003	0.868
Definite and tender	1	0.673	n/a	0.483	n/a	0.755	n/a	-0.190	n/a	0.272	n/a
**Instances of liquid stool passing per day**											
None	98	0.795	0.230	0.762	0.246	0.817	0.237	-0.034	0.002**	0.055	0.000***
1 to 3	75	0.794	0.150	0.772	0.157	0.843	0.123	-0.022	0.072	0.071	0.000***
4 to 5	46	0.729	0.183	0.704	0.179	0.761	0.193	-0.025	0.140	0.057	0.003**
6 or more	52	0.671	0.256	0.618	0.275	0.709	0.259	-0.054	0.013*	0.091	0.001**
**Blood in stools**											
None	140	0.742	0.231	0.716	0.247	0.771	0.229	-0.026	0.003**	0.055	0.000*
Streaks of blood with stools in less than half of cases	34	0.811	0.156	0.773	0.164	0.806	0.203	-0.038	0.071	0.034	0.109*
Obvious blood with most of stools	29	0.780	0.173	0.752	0.192	0.807	0.221	-0.028	0.122	0.056	0.090*
Blood alone passes	68	0.762	0.215	0.715	0.224	0.828	0.180	-0.047	0.012*	0.113	0.000*
**Anaemia**											
No	216	0.769	0.182	0.728	0.205	0.804	0.192	-0.041	0.000***	0.076	0.000***
Yes	55	0.726	0.309	0.724	0.299	0.755	0.283	-0.002	0.883	0.031	0.018*
**Colonoscopy findings**											
No SBD (not life-threatening condition, no immediate action needed) ^d^	227	0.751	0.222	0.720	0.236	0.794	0.223	-0.031	0.000***	0.074	0.000***
SBD (not life-threatening condition, immediate action needed)^e^	34	0.825	0.152	0.781	0.156	0.817	0.124	-0.044	0.049*	0.036	0.094
SBD (life-threatening condition, urgent action needed)^f^	10	0.738	0.164	0.696	0.200	0.709	0.235	-0.042	0.089	0.014	0.623

SBD: severe bowel disease

^a^ P-values derived from paired t-test for null hypothesis of difference in mean EQ-5D-5 L T1 = EQ-5D-5 L T2 and mean EQ-5D-5 L T2 = EQ-5D-5 L T3. * P-value ≤ 0.05; **p-value ≤ 0.01; *** p-value ≤ 0.001.

^b^Question answered by former smokers (*n* = 61) and current smokers (*n* = 28)

^c^ Value unavailable owing to small number of observations

^d^ Defined as colonoscopy results showing no indications of Crohn’s disease or ulcerative colitis, or polyps < 10 mm

^e^ Defined as colonoscopy results showing mild or moderate Crohn’s disease or ulcerative colitis, or polyps > 10 mm

^f^ Defined as colonoscopy results showing presence of cancerous lesion, severe Crohn’s disease or severe ulcerative colitis

Looking across characteristics (Supplementary material Table 2), statistically significantly higher EQ-5D-5 L values were reported by male participants (vs. female, *p* < 0.001) and people of ethnic background other than White British (*p* = 0.049), while significantly lower values were reported by people classified as obese (vs. normal weight, *p* = 0.018), not alcohol drinkers (vs. alcohol drinkers, *p* < 0.00), current smokers (vs. never smoked, *p* < 0.001) and people who used to smoke, or are smoking, more than 30 cigarettes a day (vs. 1–9 cigarettes a day, *p* < 0.001). In relation to symptoms, people reported a significantly lower EQ-5D-5 L value if they experienced six or more instances of liquid stool passing per day (vs. none, *p* < 0.001).

## Discussion

The increasing incidence of colorectal cancer worldwide has highlighted the need to develop and assess diagnostic technologies that are effective and represent ‘value for money’ [5]. For findings to be dependable and comprehensive, analyses need to take into account all relevant diagnostic and therapeutic components and their impact, including the effect of undergoing an invasive examination. Failing to do so effectively implies that, in economic evaluations, a colonoscopy is considered no less unpleasant than any other approach, as well as that, practically, there is no additional benefit in developing less invasive diagnostic methods. Most people with lower bowel symptoms do not have serious bowel disease [5], but if they all have colonoscopy and some associated ‘disutility’, the cumulative disutility across the population will be considerable.

It is well known that undergoing a colonoscopy examination is unpleasant, but, to the best of our knowledge, there is a lack of empirical estimates of the decrement in preference-based HRQoL that can be used in economic analyses. To quantify the effect of the procedure, we collected data at three points in time: (i) T1: before the colonoscopy, to capture a patient’s health status prior to the procedure; (ii) T2: during bowel preparation, to capture a component of the procedure that patients find to be particularly unpleasant; and, (iii) T3: shortly after colonoscopy, to capture HRQoL after the procedure. For this, we used the EQ-5D-5 L, a multi-attribute instrument that enables the calculation of utility values and, by extension, QALYs. We provide granular estimates of utility values for different patient characteristics (including sex, age group, ethnicity, BMI etc.), gastrointestinal symptoms and diagnosis.

Despite respondents presenting with gastrointestinal symptoms, the commonest response to the EQ-5D-5 L was ‘no problems’ in all dimensions (11111). At T1, the mean EQ-5D-5L value across all respondents was 0.76 (SD: 0.21), with males reporting higher values than female respondents (0.82 vs 0.71 respectively, p-value < 0.001). These values, and the fact that on average, males tend to report higher EQ-5D-5L values, are comparable to observations in the general population in England [17]. At T2, there was a notable drop in the mean EQ-5D value across all participants (-0.33, p-value < 0.001), in line with the greater number of participants reporting higher levels of anxiety. This reduction was prominent amongst females, White British and people classified as obese. At T3, after colonoscopy, there was a notable and statistically significant rebound in HRQoL compared to the previous assessment point, with the mean EQ-5D score across all patients ending up exceeding the score at baseline.

Findings were broadly in line with intuition and previous evidence. Patients with symptoms suggesting a bowel condition were anticipated to be experiencing some problems with pain/discomfort and anxiety prior to diagnosis and treatment initiation. Similarly, it is expected that pain and anxiety would have intensified on the day before the scheduled colonoscopy examination, while undergoing bowel preparation in anticipation of a hospital appointment for an invasive procedure [4]. After the procedure, at T3, EQ-5D-5 L values had rebounded and exceeded those of T2, with more patients reporting no problems with pain/discomfort or anxiety/depression than at T2. It is likely that this is due to the discomfort associated with the preparation and procedure subsiding, and it is also possible that, by this time, the anxiety associated with the prospect of undergoing an invasive procedure is allayed.

To our knowledge, our study is unique in measuring the effect of a colonoscopy using a generic multi-attribute instrument and presenting findings in terms of preference-based HRQoL (utility). Previous studies aiming to explore the impact of colonoscopy on HRQoL have done so by using instruments that do not allow the derivation of utility values [4, 18, 19], a necessary component for the calculation of QALYs in cost-utility analyses. An exception is a recent study by Bulamu et al. [20], where EQ-5D-5 L was used in addition to the cancer-specific EORTC Quality of Life Utility Measure-Core 10 dimensions (QLU-C10D). However, the aims of these study were different—to assess the sensitivity and discriminant validity of two instruments—and responses were sought well after colonoscopy had taken place (median times 38 days and 423 days after colonoscopy, for first and second assessment, respectively), rather than shortly before, during and shortly after the procedure.

Our study has certain limitations which warrant careful interpretation of its findings. First, the population of study participants presented little ethnic diversity: nearly 92% of the participants identified as White British. This is in line with the broader sample of all RECEDE participants, where 1809 out of 1978 patients (91.4%) identified as White British and reflects findings that, in the UK, ethnic minorities are more likely to be diagnosed as emergencies, rather than through the two-week-wait pathway. Given this, it may be reasonable to consider EQ-5D-5 L values derived in this study to be largely applicable to a White British population, though there is no obvious reason why responses of participants of other ethnicities might be systematically different, especially when it comes to dimensions of interest in the EQ-5D-5 L such as mobility, self-care, usual activities, pain/discomfort, anxiety/depression. Secondly, findings reported here are based on a complete-case analysis; participants with incomplete data (most commonly not having returned a complete EQ-5D-5 L questionnaire at a single assessment point) were, pragmatically, excluded from the analysis. Methods are available to impute missing data and, while these can increase the number of responses, they require ascertaining the mechanism that caused missingness and add an additional level of uncertainty [21]. Thirdly, the degree to which a generic HRQoL such as the EQ-5D-5 L is sensitive enough to capture the effect of a procedure on HRQoL is debatable. While, it is expected that condition-specific instruments might be better at detecting changes in this population [20], measures derived from non-preference-based HRQoL scales are not appropriate for use in economic evaluations [10], thus a balance needs to be struck between sensitivity and useability. Last, it can be argued that the most accurate indication of the HRQoL decrement due to undergoing a colonoscopy would be obtained by participants completing the EQ-5D-5 L while they are physically undergoing the examination. As this is, for obvious reasons, not advisable, the thirds assessment took place after the procedure (within 24 h). It is likely that, at the end of the procedure, patients are given an (informal) indication of the colonoscopy findings, thus it is possible that a drop in anxiety levels at T3 is, at least partially, due to participants receiving reassurance that SBD was not identified. Nonetheless, looking across patients who were and were not diagnosed with SBD showed EQ-5D-5 L scores to follow a similar, increasing trajectory between T2 and T3, though this increase was not statistically significant in patients diagnosed with SBD (*p* = 0.623).

## Conclusions

People experiencing gastrointestinal symptoms referred for further investigation report some issues with pain/discomfort and anxiety/depression and a mean utility value equivalent to this in the general population. The utility values decreases statistically significantly while undergoing bowel preparation and rebounds after colonoscopy is completed. Utility values vary with patient characteristics, symptoms and diagnoses, and these values can be used to quantify and reflect the HRQoL associated with undergoing a colonoscopy in economic evaluations.

### Electronic supplementary material

Below is the link to the electronic supplementary material.

Supplementary Material 1

## Data Availability

No datasets were generated or analysed during the current study.
