# Supplementary material for: Impact of colonoscopy on health-related quality of life: findings from the RECEDE study
Source: Health Qual Life Outcomes. 2024 Jun 26;22:49. doi: 10.1186/s12955-024-02262-x (PMC11210081; doi:10.1186/s12955-024-02262-x)
Supplement: Supplementary file 1 — Supplementary Material 1 [file 12955_2024_2262_MOESM1_ESM.docx]

Supplementary Material Table 1. Correlation between EQ VAS and EQ-5D-5L values.

|  | **EQ VAS at T1** | **EQ VAS at T2** | **EQ VAS at T3** |  | **EQ-5D-5L value at T1** | **EQ-5D-5L value at T2** | **EQ-5D-5L value at T3** |
| --- | --- | --- | --- | --- | --- | --- | --- |
| **EQ VAS T1** | 1 |  |  |  |  |  |  |
| **EQ VAS T2** | 0.8275*** | 1 |  |  |  |  |  |
| **EQ VAS T3** | 0.8053*** | 0.8302*** | 1 |  |  |  |  |
|  |  |  |  |  |  |  |  |
| **EQ-5D value T1** | 0.7093*** | 0.6421*** | 0.6361*** |  | 1 |  |  |
| **EQ-5D value T2** | 0.6602*** | 0.7252*** | 0.6036*** |  | 0.8593*** | 1 |  |
| **EQ-5D value T3** | 0.6564*** | 0.6415*** | 0.7404*** |  | 0.8283*** | 0.8033*** | 1 |
| * P≤0.05; ** P≤0.01; ***P≤0.001 | | | | | | | |

Supplementary Material Table 2. Comparison between categories of factor variables (demographic characteristics, referral symptoms, diagnosis).

|  |  | **T1** | | **T2** | | **T3** | |
| --- | --- | --- | --- | --- | --- | --- | --- |
| **EQ-5D-5L index values** | **N** | **Mean** | **p-value^a^** | **Mean** | **p-value^a^** | **Mean** | **p-value^a^** |
| **Age group** |  |  |  |  |  |  |  |
| 20-39 (RC) | 17 | 0.732 | - | 0.678 | - | 0.791 | - |
| 40-59 | 101 | 0.761 | 0.527 | 0.726 | 0.429 | 0.784 | 0.870 |
| 60-79 | 147 | 0.759 | 0.538 | 0.730 | 0.393 | 0.799 | 0.851 |
| over 80 | 6 | 0.852 | 0.073 | 0.803 | 0.179 | 0.835 | 0.481 |
| Overall^b^ |  |  | 0.327 |  | 0.612 |  | 0.802 |
| **Sex** |  |  |  |  |  |  |  |
| Female (RC) | 142 | 0.708 | - | 0.662 | - | 0.736 | - |
| Male | 129 | 0.817 | 0.000*** | 0.798 | 0.000*** | 0.857 | 0.000*** |
| Overall^b^ |  |  | 0.000*** |  | 0.000*** |  | 0.000*** |
| **Ethnicity** |  |  |  |  |  |  |  |
| Other ethnicity (RC) | 15 | 0.822 | - | 0.722 | - | 0.791 | - |
| White British | 256 | 0.756 | 0.049* | 0.816 | 0.029* | 0.839 | 0.166 |
| Overall^b^ |  |  | 0.049* | 0.000 | 0.029* |  | 0.166 |
| **BMI group** |  |  |  |  |  |  |  |
| Healthy weight (RC) | 78 | 0.778 | - | 0.753 | - | 0.829 | - |
| Underweight | 1 | 0.893 | 0.000*** | 0.793 | 0.091 | 0.793 | 0.107 |
| Overweight | 113 | 0.787 | 0.777 | 0.756 | 0.910 | 0.811 | 0.537 |
| Obese | 79 | 0.702 | 0.018 | 0.658 | 0.008** | 0.735 | 0.006** |
| Overall^b^ |  |  | 0.000*** |  | 0.000*** |  | 0.025* |
| **Alcohol drinking** |  |  |  |  |  |  |  |
| Alcohol drinker (RC) | 168 | 0.792 | - | 0.761 | - | 0.823 |  |
| Non-drinker | 103 | 0.708 | 0.003** | 0.671 | 0.003** | 0.746 | 0.008** |
| Overall^b^ |  |  | 0.003** |  | 0.003** |  | 0.008** |
| **Smoking** |  |  |  |  |  |  |  |
| Non-smoker (RC) | 182 | 0.771 | - | 0.734 | - | 0.806 | - |
| Former Smoker | 61 | 0.804 | 0.200 | 0.765 | 0.25 | 0.819 | 0.649 |
| Current Smoker | 28 | 0.589 | 0.006** | 0.596 | 0.049* | 0.657 | 0.023** |
| Overall^b^ |  |  | 0.010** |  | 0.067 |  | 0.067 |
| **Cigarettes per day ^c^** |  |  |  |  |  |  |  |
| 1 to 9 (RC) | 20 | 0.664 | - | 0.683 | - | 0.741 | - |
| 10 to 19 | 40 | 0.733 | 0.414 | 0.688 | 0.951 | 0.745 | 0.963 |
| 20 to 29 | 22 | 0.748 | 0.336 | 0.727 | 0.588 | 0.779 | 0.646 |
| More than 30 | 7 | 0.926 | 0.001** | 0.890 | 0.009** | 0.942 | 0.009** |
| Overall^b^ |  |  | 0.000*** |  | 0.000*** |  | 0.000*** |
| **Abdominal mass** |  |  |  |  |  |  |  |
| None (RC) | 268 | 0.760 | - | 0.727 | - | 0.794 | - |
| Definite | 2 | 0.856 | 0.000*** | 0.798 | 0.000*** | 0.795 | 0.950 |
| Definite and tender | 1 | 0.673 | 0.000*** | 0.483 | 0.000*** | 0.755 | 0.003** |
| Overall^b^ |  |  | 0.000*** |  | 0.000*** |  | 0.000*** |
| **Instances of liquid stool passing per day** |  |  |  |  |  |  |  |
| None (RC) | 98 | 0.795 | - | 0.762 | - | 0.817 | - |
| 1 to 3 | 75 | 0.794 | 0.964 | 0.772 | 0.756 | 0.843 | 0.364 |
| 4 to 5 | 46 | 0.729 | 0.063 | 0.704 | 0.128 | 0.761 | 0.163 |
| 6 or more | 52 | 0.671 | 0.003** | 0.618 | 0.002** | 0.709 | 0.013 |
| Overall^b^ |  |  | 0.000*** |  | 0.001** |  | 0.001** |
| **Blood in stools** |  |  |  |  |  |  |  |
| None (RC) | 140 | 0.742 | - | 0.716 | - | 0.771 | - |
| Streaks of blood with stools in less than half of cases | 34 | 0.811 | 0.042* | 0.773 | 0.114 | 0.806 | 0.394 |
| Obvious blood with most of stools | 29 | 0.780 | 0.314 | 0.752 | 0.383 | 0.807 | 0.433 |
| Blood alone passes | 68 | 0.762 | 0.558 | 0.715 | 0.974 | 0.828 | 0.054 |
| Overall^b^ |  |  | 0.229 |  | 0.380 |  | 0.277 |
| **Anaemia** |  |  |  |  |  |  |  |
| No (RC) | 216 | 0.769 | - | 0.728 | - | 0.804 | - |
| Yes | 55 | 0.726 | 0.364 | 0.724 | 0.933 | 0.755 | 0.263 |
| Overall^b^ |  |  | 0.364 |  | 0.933 |  | 0.263 |
| **Colonoscopy findings** |  |  |  |  |  |  |  |
| Not life-threatening condition, no immediate action needed ^d^ (RC) | 227 | 0.751 | - | 0.720 | - | 0.794 | - |
| Not life-threatening condition, immediate action needed ^e^ | 34 | 0.825 | 0.021* | 0.781 | 0.046* | 0.817 | 0.393 |
| Life-threatening condition, urgent action needed ^f^ | 10 | 0.738 | 0.819 | 0.696 | 0.721 | 0.709 | 0.289 |
| Overall^b^ |  |  | 0.059 |  | 0.118 |  | 0.356 |
| ^a^ P-values derived from ordinary least squares regression using bootstrap resampling. Null hypothesis: coefficient of factor variable is equal to zero. * P-value ≤ 0.05; **p-value ≤ 0.01; *** p-value ≤ 0.001.  ^b^ Wald joint test, testing that all coefficients of a factor variable are equal to zero. ^c^ Question answered by former smokers (n=61) and current smokers (n=28). ^d^ Defined as colonoscopy results showing no indications of Crohn's disease or ulcerative colitis, or polyps < 10mm.  ^e^ Defined as colonoscopy results showing mild or moderate Crohn's disease or ulcerative colitis, or polyps > 10mm.  ^f^ Defined as colonoscopy results showing presence of cancerous lesion, severe Crohn's disease or severe ulcerative colitis.  SBD: severe bowel disease.  RC: reference category. | | | | | | | |
